# Modulatory Effects of Hypertension on Aging‐Related White Matter Hyperintensities: A Comparative Study Among Stroke Patients and Stroke‐Free Community‐Based Cohort

**DOI:** 10.1111/jch.70002

**Published:** 2025-02-28

**Authors:** Joseph A. Ackah, Du Heng, Xuelong Li, Lu Zheng, Jason Tsz Lok Chan, Michael Lung Cheung Lo, Jun Hu, Xiangyan Chen

**Affiliations:** ^1^ Department of Health Technology and Informatics The Hong Kong Polytechnic University Kowloon Hong Kong SAR China; ^2^ Department of Neurology, Institute of Neuroscience, Key Laboratory of Neurogenetics and Channelopathies of Guangdong Province and the Ministry of Education of China The Second Affiliated Hospital Guangzhou Medical University Guangzhou China; ^3^ Department of Neurology The Third Affiliated Hospital of Sun Yat‐sen University Guangzhou China; ^4^ Department of Neurology Peking University Shenzhen Hospital Shenzhen China

**Keywords:** aging, cerebral small vessel diseases, hypertension, modulation, stroke, white matter hyperintensities

## Abstract

The increased vulnerability of the aging human brain to hypertension‐induced neurovascular impairments, including cerebral small vessel diseases (SVD), marked by MRI‐visible white matter hyperintensities (WMH), is well recognized. We examined WMH burdens between stroke patients and stroke‐free participants across three age groups and explored patterns of modifiable risk factors, specifically the modulating effects of hypertension on WMH burden, providing insights for potential therapeutic interventions. This study comprised one hospital‐based cohort of 254 stroke patients and another community‐based cohort of 254 stroke‐free normative participants. Clinical variables were obtained consecutively, and MRI neuroimaging classified WMH as absent, mild, moderate, or severe. A step‐by‐step statistical analysis was performed to explore the said gaps. There were 508 participants (mean age 63.5 ± 8.9 years) with 285 males. A similar prevalence but different WMH burden was recorded between stroke and normative cohorts across different age groups. The modulating effect of hypertension on WMH severity varied across age groups and is greater in middle‐aged adults; intriguingly, this effect diminished in elderly adults (*b* = −0.882, 95%CI [−1.591, –0.172], *t* = −2.442, *p* = 0.015). It was shown that, in a non‐uniform fashion across different age groups, hypertension is a culprit risk factor for exacerbating WMH severity, and middle‐aged adults are the most vulnerable. While the elevation of systolic blood pressure predisposes adults to brain white matter deterioration, the decline in diastolic blood pressure suggests a protective role. Recognizing hypertension as a modifiable risk factor and understanding the aging‐related changes in blood pressure patterns open avenues for developing age‐specific strategies for the mitigation and management of WMH progression.

## 1 Background

The increased susceptibility of the human aging brain to hypertension‐induced neurovascular dysfunction and associated cerebrovascular impairments has been widely discussed [1, 2, 3, 4]. In particular, endothelial dysfunction and vessel wall stiffness are key vascular mechanisms linked to brain structural alterations in both hypertension and advanced aging [5, 6]. Although it is generally considered that aging correlates linearly with increased blood pressure (BP), evidence shows that the patterns of systolic and diastolic BP change with aging [7]. Elevated systolic and decreased diastolic blood pressure (DBP) [8], commonly observed in individuals aged 50 and above, leads to increased pulse pressure, which indicates significant vascular risk [9, 10]. The uncertainty surrounding the clinical value of age‐related shifts in BP patterns for predicting and managing stroke risk and cognitive decline in patients with cerebral white matter changes may be influenced by the modulating effects of hypertension across different age groups [11, 12, 13]. These changes in BP are particularly relevant when considering their impact on white matter hyperintensities (WMH) of presumed vascular origin, a common neuroimaging marker of cerebral small vessel disease (SVD) [2, 4]. Current evidence connects WMH to stroke severity [4], risk of stroke recurrence [14], cognitive decline [15], gait and balance disturbances [16], neuropsychiatric disorders [17], and high risks of mortality [18], all of which affect the quality of life of current aging populations. Despite this, the pathophysiology and the patterns of WMH severity burden between people with stroke and stroke‐free populations have not been distinctively delineated [19, 20, 21].

While aging and hypertension, marked by increased systolic and diastolic BP, are recognized as prevalent cerebrovascular risk factors [22, 23], the modulating influences of these factors on WMH burden among middle‐aged, older adults, and elderly adults have been consistently overlooked. It is also noted that, although evidence points to aging‐related shifts in BP patterns, previous studies have predominantly examined the impact of aging on brain white matter changes in a non‐categorized linear manner [12, 24–26]. One can posit that exploring such relationships across different categories of age groups in a cohort of stroke patients and stroke‐free individuals could reveal clinically significant patterns that enhance precision therapy and prevention strategies. The literature hints that the lack of critical comparative investigations incorporating age‐stratified risk assessments of SVDs is partly responsible for the inconsistent outcomes in cerebral SVD pathophysiological investigations [21, 27]. The increasing prevalence of cerebrovascular diseases, coupled with the complex interplay of risk factors like aging, hypertension, and lifestyle changes [28, 29], underscores the need to deepen our understanding of these dynamics. It is, therefore, essential to provide more current and statistically robust evidence to better explore these underexamined areas.

The aim of this study was to examine how the severity of WMH varies with age in both stroke patients and individuals without stroke, and to explore how modifiable risk factors, particularly hypertension, affect the burden of WMH in different age groups. A sub‐focus was to explore the mediation effects of BP on the relationship between aging and WMH burden.

## 2 Methods and Materials

### 2.1 Study Design and Population

This study combined two cohorts: a hospital‐based cohort with 265 stroke patients and a community‐based cohort with 268 stroke‐free normative participants. Combining the two cohorts was aimed at elucidating the distribution of WMH burden within a combined cohort of stroke patients and community‐based stroke‐free individuals, enhancing the generalizability of the study findings and their applicability to a broad range of people in both clinical and community settings. The **normative** cohort was comprised of Chinese community‐based occupants with no history of stroke, dementia, substance abuse, or diagnosed neuropsychiatric condition using simple random sampling techniques from November 2022 and December 2023. The **stroke** cohort was sampled from the electronic medical database of patients admitted to Peking University Shenzhen Hospital's stroke center between November 2022 and August 2023. The **stroke cohort** was formed in adherence to the inclusion criteria set priorly: (1) Chinese patients aged 40–90 years who were diagnosed or had an acute ischaemic stroke or transient ischaemic attack (TIA); (2) brain magnetic resonance imaging (MRI) with fluid‐attenuated inversion recovery (FLAIR) sequence.

The researchers excluded participants in accordance with the following criteria: (1) patients with contraindications to MRI; (2) patients with other conditions that mimic or cause white matter lesions, including multiple sclerosis, vasculitis, or connective tissue diseases; (3) patients with brain tumors and traumatic head injuries; and (4) patients with incomplete clinical data and poor imaging data. The research protocol was evaluated and approved by the Ethics Committee of the Hong Kong Polytechnic University and the Clinical Research Ethics Committee of the Peking University Shenzhen Hospital. All participants provided written informed consent prior to the commencement of the study. The study was reported according to the STROBE guidelines [30].

### 2.2 Clinical Data Collection

**FIGURE 1:**
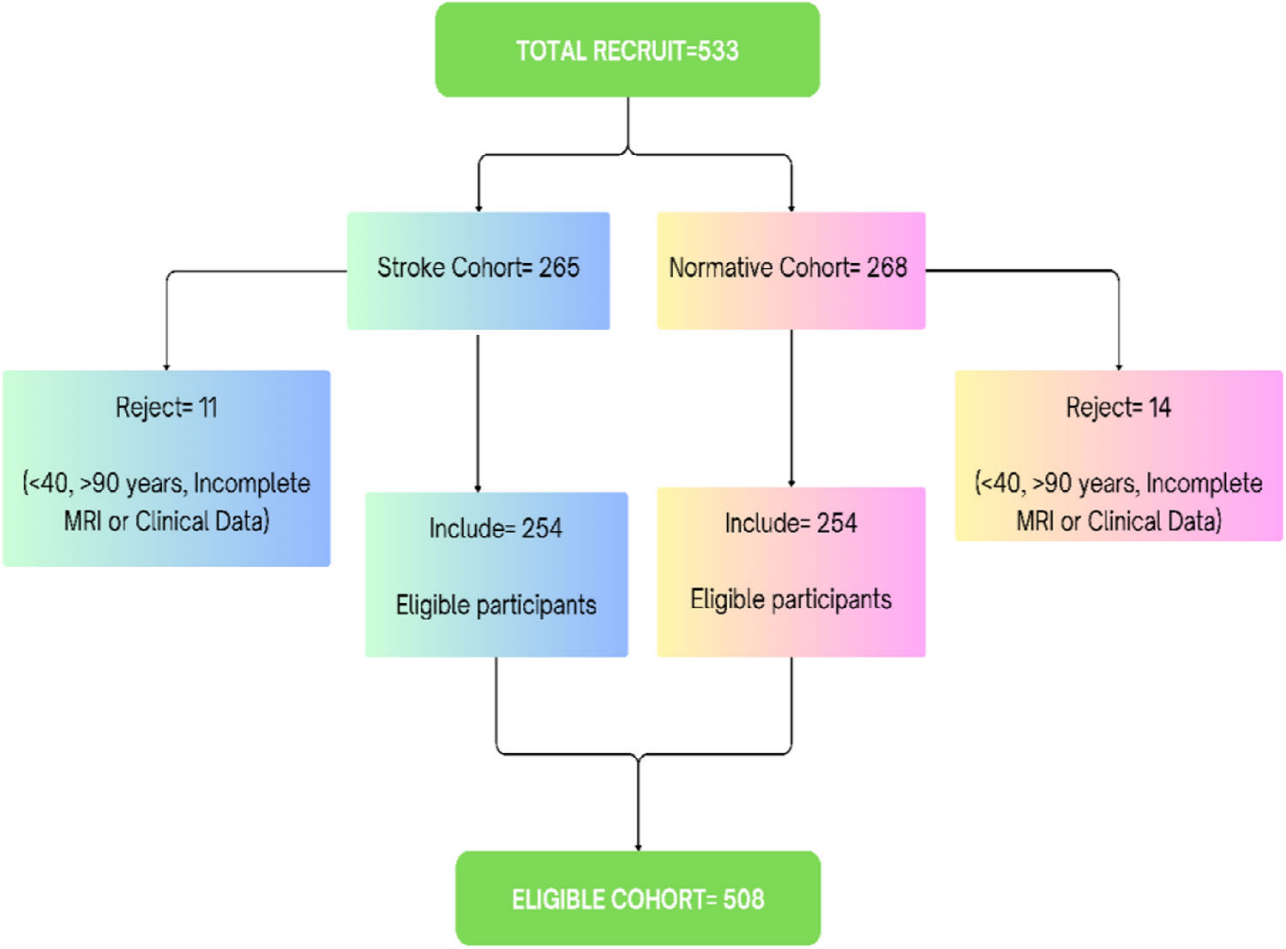
Participant selection.

Clinical variables recorded include age, gender, BP, history of hypertension, diabetes, smoking, drinking, hyperlipidemia, and a history of antihypertensive drugs. Participants were categorized by age into **middle‐aged adults (40–59 years), older adults (60–79 years), and elderly adults (80–90 years)**. Hypertension was defined based on systolic blood pressure (SBP) ≥ 140 mmHg and/or diastolic blood pressure (DBP) ≥ 90 mmHg [31]. Diabetes mellitus was detected at fasting glucose ≥ 7.0 mmol/L and/or random blood glucose ≥ 11.1 mmol/L [32]. Body mass index (BMI) was calculated from the ratio of weight and height [33]. The classifications of smoking and drinking were based on patient self‐report and were both defined as continuous or cumulative smoking and consumption of alcohol for more than 6 months in a lifetime, respectively, as adopted by Zheng and colleagues [34]. Data protection protocols and confidentiality were strictly adhered to. Figure 1 illustrates the selection process of participants.

### 2.3 Neuroimaging Acquisition and Assessment

MRI imaging acquisition protocols and the validated visual assessment scales [35] adopted for evaluating WMH in the **stroke cohort** have been previously reported [36]. For the community‐based **normative cohort**, a 3.0‐Tesla MRI (Siemens Medical System, Erlangen, Germany) with a 16‐channel head coil was used for brain scans at the University Research Facility in Behavioural and Systems Neuroscience (UBSN), the Hong Kong Polytechnic University. The protocol involved a T2‐weighted fluid‐attenuated inversion recovery (FLAIR) sequence: 0.9 mm sagittal slices, TE = 395 ms, TR = 7000 ms, field of view = 230 mm × 230 mm, acceleration factor = 6, auto voxel size = 0.9 × 0.9 × 0.9 mm^3^, acquisition time = 3.46 min.

### 2.4 White Matter Hyperintensities Grading

**FIGURE 2:**
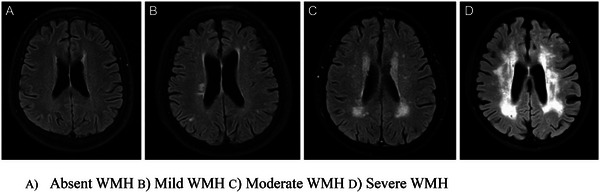
FLAIR images of different WMH severity. (A) Absent WMH, (B) mild WMH, (C) moderate WMH, and (D) severe WMH. WMH, white matter hyperintensities.

Brain MRI image assessment was independently conducted using imaging analysis software (OsiriX DICOM Viewer‐Pixmeo SARL, Bernex, Switzerland, version 13.0.2) by two experienced neuroimaging clinical investigators (J.A.A. and H.D.) who were blinded to the clinical data. Prior to WMH assessment, diffusion‐weighted imaging (DWI) and apparent diffusion coefficient (ADC) were used to rule out acute infarct lesions. WMH severity scores and overall burden were graded in accordance with a previously published and validated 8‐grade scale [35]. Four categories for WMH burden based on the severity scores were established as follows: absent (grade 0), mild (grade 1–2), moderate (grade 3–5), and severe (grade 6–8), as shown in Figure 2. The inter‐rater reliabilities, as assessed by Cohen's kappa analysis, were excellent (>0.8).

### 2.5 Statistical Analysis

The current 29.0 version of IBM SPSS was used for statistical analysis. Continuous quantitative variables were presented as mean ± standard deviation (SD), and categorical variables were presented as frequencies and percentages. A *t*‐test, Mann–Whitney *U*, and Kruskal–Wallis *H* (with post hoc analysis) tests were used to compute significant differences within continuous variables. Relationships between categorical variables were assessed using Fisher's exact test. Multivariate ordinal regression analysis was performed to explore the independent predictors of WMH burden. Following the assessment of modifiable risk factors, a moderation analysis was performed to evaluate the modulating effects of significant vascular risk factors on WMH burden across middle‐aged adults, older adults, and elderly adults. A mediation analysis evaluated the effects of BP on the relationship between age and WMH burden. A two‐tailed *p* value < 0.05 was considered statistically significant.

## 3 Results

### 3.1 Participant's Characteristics

Out of the initial pool of 533 candidates (265 for stroke and 268 for normative), a total of 25 candidates (11 stroke and 14 stroke‐free normative) were excluded for (1) incomplete MRI and clinical data, (2) unavailable FLAIR sequence, (3) poor image quality, and (4) ages of <40 and >90 years. Finally, 508 participants were retained for the analysis, with each cohort comprising 254 members each for stroke and normative cohorts, respectively. Among the total 508 participants, with a mean age of 63.5 ± 8.9 years, the mean systolic BP was 142.95 ± 22.99 mmHg, and the mean diastolic BP was 84.16 ± 13.86 mmHg. Two hundred and eighty‐five (56.1%) were males, 118 (23.2%) were smokers, 102 (20.1%) were drinkers, 111 (21.9%) had diabetes, 249 (49.0%) had hypertension, and 145 (28.5%) had hyperlipidemia. A total of 190 (37.4%) out of 508 participants were using antihypertensive medications, with a significant portion of them, 61.6% (117 individuals), being stroke patients.

**FIGURE 3:**
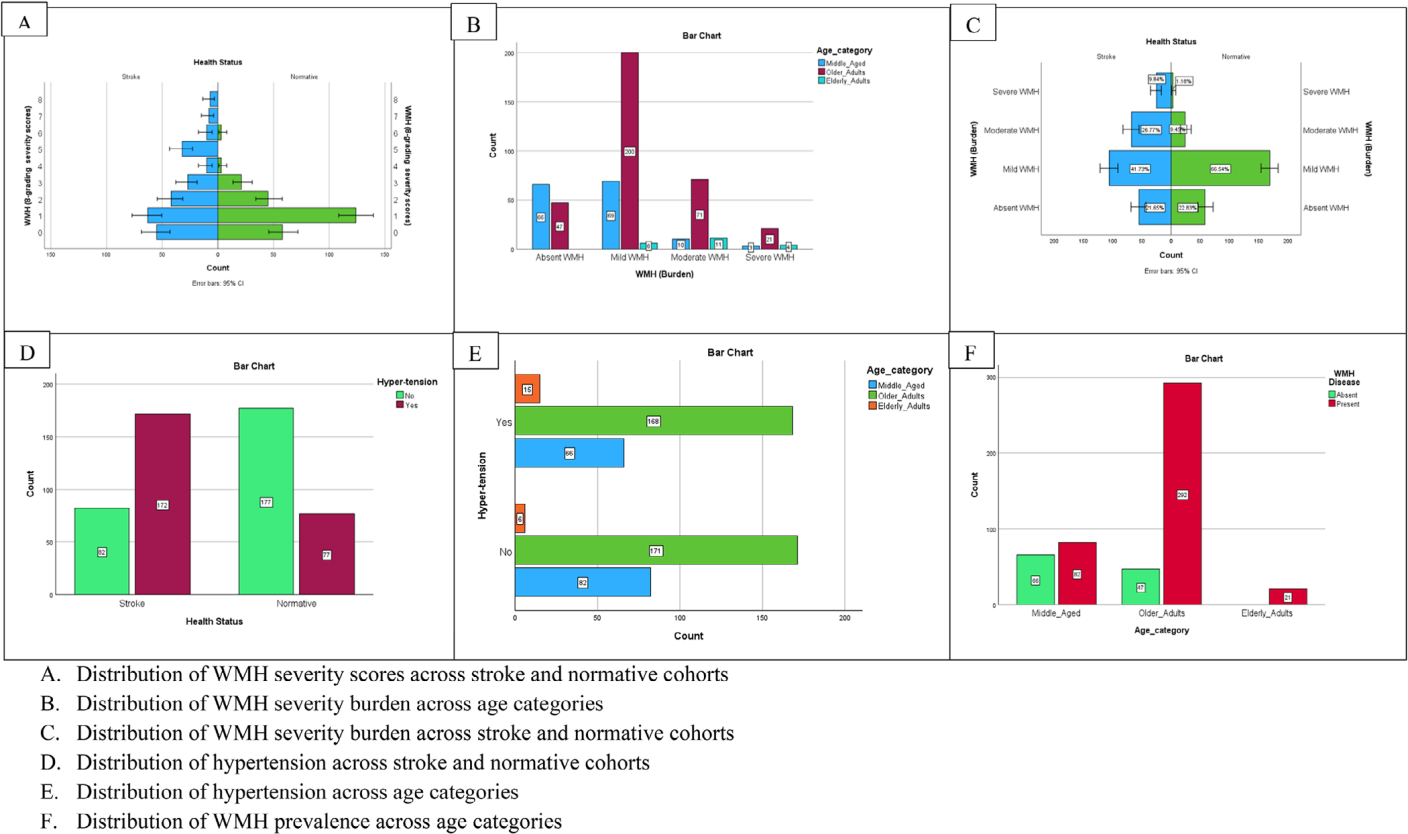
Distribution of WMH severity burden. (A) Distribution of WMH severity scores across stroke and normative cohorts. (B) Distribution of WMH severity burden across age categories. (C) Distribution of WMH severity burden across stroke and normative cohorts. (D) Distribution of hypertension across stroke and normative cohorts. (E) Distribution of hypertension across age categories. (F) Distribution of WMH prevalence across age categories. WMH, white matter hyperintensities.

There were 148 (29.1%) middle‐aged adults, 339 (66.7%) older adults, and 21 (4.1%) elderly adults. WMH was present among 82 (55.4%) of the middle‐aged adults, 292 (86.1%) of the older adults, and 21 (100%) of the elderly adults. Individuals with WMH tended to be older than those without WMH, with most falling into the older adult age range of **60 to 79 years**. Hypertension was significantly more prevalent among individuals with WMH compared to those without WMH. However, the distribution of diabetes, hyperlipidemia, smoking, drinking, and gender was similar between the two groups. Among the 395 individuals with WMH, 156 (39.5%) were on antihypertensive medications. The demographic and clinical characteristics are shown in Table 1. The graphical representations of major statistical outcomes are illustrated in Figure 3.

**TABLE 1:** Baseline characteristics of study participants on the occurrence of WMH.

	Total population (*n* = 508)	Normative cohort (*n* = 254)	Stroke cohort (*n* = 254)	WMH present (*n* = 395)	WMH absent (*n* = 113)	*p* value
Normative status, *n* (%)	—	—	—	196 (49.6)	58 (51.3)	0.603
Gender (male), *n* (%)	285 (56.1)	114 (44.9)	171 (67.3)	220 (55.7)	65 (57.5)	0.748
Age (mean ± SD, years)	63.50 ± 8.90	63.62 ± 6.45	63.37 ± 10.86	65.25 ± 8.33	57.34 ± 8.20	<0.001
Middle‐aged, *n* (%)	148 (29.1)	58 (22.8)	90 (35.4)	82 (20.8)	66 (58.4)	<0.001
Older adults, *n* (%)	339 (66.7)	193 (76.0)	146 (57.5)	292 (73.9)	47 (41.6)	<0.001
Elderly adults, *n* (%)	21 (4.1)	3 (1.2)	18 (7.0)	21 (5.3)	0 (0.00)	<0.001
SBP (mean ± SD, mmHg)	142.95 ± 22.99	129.69 ± 16.66	156.20 ± 20.69	144.89 ± 22.45	136.17 ± 23.64	<0.001
DBP (mean ± SD, mmHg)	84.16 ± 13.86	78.60 ± 9.59	89.71 ± 15.19	84.42 ± 13.82	83.29 ± 14.01	0.452
Antihypertensive drug (Yes)	190 (37.4)	73 (28.7)	117 (46.1)	156 (39.5)	34 (30.09)	0.078
Smoking, *n* (%)	118 (23.2)	13 (5.1)	105 (41.3)	91 (23.0)	27 (23.9)	0.900
Drinking, *n* (%)	102 (20.1)	24 (9.4)	78 (30.7)	80 (20.3)	22 (19.5)	0.895
Diabetes, *n* (%)	111 (21.9)	28 (11.0)	83 (32.7)	89 (22.5)	22 (19.5)	0.521
Hypertension, *n* (%)	249 (49.0)	77 (30.3)	172 (67.7)	208 (52.7)	41 (36.3)	0.003
Hyperlipidemia, *n* (%)	145 (28.5)	97 (38.2)	48 (18.9)	196 (49.6)	58 (51.3)	0.409

*Notes*: *p* values indicate the statistical differences between participants with and without WMH.

Abbreviations: SD, means standard deviation; WMH, white matter hyperintensities.

### 3.2 Distribution of WMH Burden

Out of the combined 508‐cohort, 395 (77.8%) participants had WMH, with a similar prevalence of 196 (49.6%) and 199 (50.4%) (*p* > 0.05) recorded among stroke‐free normative participants and stroke patients, respectively. The degree of WMH burden among the entire 508‐cohort was distributed as 113 (22.2%) for absent, 275 (54.1%) for mild, 92 (18.1%) for moderate, and 28 (5.5%) for severe. Further analysis showed that 388 participants exhibited **absent‐to‐mild** WMH burdens, whereas 120 participants exhibited **moderate‐to‐severe** WMH burdens. Of the 120 recorded cases of moderate‐to‐severe WMH burden, the majority, 93 (77.5%), were stroke patients, while 27 (22.5%) were normative participants. A Mann–Whitney *U* test indicated a statistically significant greater severity burden of WMH in stroke patients compared to normative participants (U = 23 497.50, Z = 5.48, *p* < 0.001, *r* = 0.24).

Individuals with moderate‐to‐severe WMH burden were relatively older and exhibited higher systolic and diastolic BP levels compared to those with absent‐to‐mild burdens of WMH. The 120 participants who exhibited moderate‐to‐severe WMH burden (M = 159.88, SD = 21.75) compared to the 388 participants with absent‐to‐mild burdens of WMH (M = 139.57, SD = 22.32) demonstrated significantly higher systolic BP scores, *t*(506) = 6.173, *d* = 0.645, *p* < 0.001. Additionally, the 120 participants who exhibited moderate‐to‐severe WMH burden (M = 87.33, SD = 15.38) compared to the 388 participants with absent‐to‐mild burdens of WMH (M = 83.18, SD = 13.22) demonstrated significantly higher diastolic BP scores, *t*(506) = 2.894, *d* = 0.302, *p* < 0.001. Of the 190 individuals taking antihypertensive drugs, 58 (30.5%) participants exhibited nearly half (48.33%) of moderate‐to‐severe WMH burdens. The statistical distribution of the WMH burden is shown in Table 2.

**TABLE 2:** Characteristic distribution of WMH burden in total population.

	Absent/mild WMH (*n* = 388)	Moderate/severe WMH (*n* = 120)	*p* value
Normative status, *n* (%)	227 (58.5)	27 (22.5)	<0.001
Gender (male), *n* (%)	217 (55.9)	74 (60.67)	0.712
Age (mean ± SD, years)	61.71 ± 8.17	69.27 ± 8.85	<0.001
SBP (mean ± SD, mmHg)	139.57 ± 22.32	153.88 ± 21.75	<0.001
DBP (mean ± SD, mmHg)	83.18 ± 13.22	87.33 ± 15.38	0.004
Antihypertensive drug (yes)	132 (32.02)	58 (48.33)	0.005
Smoking, *n* (%)	58 (14.95)	33 (27.50)	0.217
Drinking, *n* (%)	74 (19.07)	28 (23.33)	0.301
Diabetes, *n* (%)	78 (20.10)	33 (27.50)	0.100
Hypertension, *n* (%)	160 (41.24)	89 (74.17)	<0.001
Hyperlipidemia, *n* (%)	133 (34.28)	32 (26.67)	0.645

Abbreviations: SD, means standard deviation; WMH, white matter hyperintensities.

### 3.3 Cohort‐Specific Variations in WMH Burden Across Age Groups

A Kruskal–Wallis *H* test revealed a significant difference in the mean ranks (179.67 for middle‐aged adults, 278.81 for older adults, and 389.40 for elderly adults) of WMH severity burden between the different age groups, *H* (2) = 70.2, *p* < 0.001. After a Bonferroni adjustment to the alpha level (0.05/3 = 0.0167), a Mann–Whitney for post hoc analysis revealed statistically significant differences in mean ranks among each pairing (*p* < 0.001) in the entire cohort. Furthermore, there were statistical differences in mean ranks of WMH severity burden exclusively observed across the different age groups in the respective cohorts of normative and stroke patients. Notably, each cohort (stroke or normative) revealed statistically significant differences in mean ranks among middle‐aged and older adults (*p* < 0.001) but not between older adults and elderly adults. However, in the normative group, no significant difference in WMH severity burden was noted between middle‐aged adults (mean rank = 30.23) and elderly adults (mean rank = 45.83), *p* = 0.108. Table 3 detailed the statistics with post hoc analysis.

**TABLE 3:** Cohort‐specific variations in WMH burden across age groups.

Population	*H*‐test	Age category	*N*	WMH Severity Mean Rank	U‐post hoc	*p* value
Normative 254 cohort	17.386					<0.001
		a‐b	251	(94.22–135.55)	3754.0	<0.001
		a‐c	61	(30.23–45.83)	42.5	0.108
		b‐c	196	(98.23–115.83)	237.50	0.564
Stroke 254 cohort	56.851					<0.001
		a‐b	236	(80.70–141.80)	3168.0	<0.001
		a‐c	108	(47.87–87.67)	213.0	<0.001
		b‐c	164	(79.57–106.28)	886.0	0.022
Total population	70.204					<0.001
		a‐b	487	(176.91–273.29)	15 156.0	<0.001
		a‐c	169	(77.26–139.52)	409.0	<0.001
		b‐c	360	(175.52–260.88)	1871.50	<0.001

*Notes*: a‐b = middle‐aged against older adults; a‐c = middle‐aged against elderly adults; b‐c = older against elderly adults.

Abbreviation: WMH, white matter hyperintensities.

### 3.4 Predicting WMH Severity Based on Vascular Risk Profile

A multivariate ordinal regression model showed that WMH burden was regressed on predicting variables of age, hypertension, gender, health status, smoking, drinking, diabetes, and hyperlipidemia (*p* < 0.001) in the entire cohort. Coefficients were further assessed to ascertain that only age (OR = 1.115; 95% CI, 1.093–1.139; *p* < 0.001) and hypertension (OR = 2.069; 95% CI, 1.452–2.950; *p* < 0.001) were independently associated with WMH burden. A similar occurrence was exclusively observed in the respective cohorts of normative and stroke cohorts, as shown in Table 4. The regression model indicates no issues with collinearity between age and hypertension. This is supported by the variance inflation factor (VIF) values of 1.004 for both age and hypertension, as well as tolerance values of 0.996 for each variable. These results suggest that collinearity is not a concern in this analysis, as shown in the Supplementary Table.

**TABLE 4:** Risk profile for WMH severity (Multivariate ordinal regression).

	OR	95% CI (lower)	95% CI (upper)	*p* value
Normative				
Age (years)	1.111	1.069	1.154	<0.001
Age (years)*	1.093	1.051	1.138	<0.001
Hypertension	3.540	2.104	5.954	<0.001
Hypertension*	2.683	1.533	4.697	<0.001
Gender (2)	1.037	0.631	1.702	0.889
Smoking	0.862	0.279	2.667	0.797
Drinking	1.680	0.735	3.846	0.219
Diabetes	1.026	0.443	2.377	0.952
Hyperlipid	1.010	0.590	1.728	0.971
Stroke				
Age (years)	1.100	1.076	1.126	<0.001
Age (years)*	1.111	1.084	1.140	<0.001
Hypertension	1.665	1.042	2.659	0.033
Hypertension*	2.037	1.244	3.333	0.005
Gender (2)	1.088	0.609	1.946	0.775
Smoking	0.995	0.568	1.745	0.986
Drinking	1.513	0.862	2.660	0.149
Diabetes	1.032	0.689	1.547	0.878
Hyperlipid	0.951	0.528	1.712	0.866
Total population				
Age (years)	1.115	1.093	1.139	<0.001
Health Status*	2.038	1.363	3.047	<0.001
Gender (2)	1.063	0.734	1.539	0.747
Smoking	0.943	0.588	1.511	0.808
Drinking	1.513	0.960	2.385	0.074
Diabetes	1.032	0.689	1.547	0.878
Hypertension	2.069	1.452	2.950	<0.001
Hyperlipidem	0.134	1.493	0.884	2.520

*Note*: b. Ordinal regression odds for WMH severity.*Adjusted for confounders.

### 3.5 Moderation and Mediation Effects on WMH Severity Burden

The moderation analysis revealed hypertension as the vascular risk factor with a significant modulating influence on WMH burden across different age groups. The model demonstrated a significant fit, capable of accounting for potential variations in the moderation effect across both categorized and non‐categorized age groups (*p* < 0.001 for both). In the non‐categorized age groups, a non‐significant moderation by the effect of hypertension was shown in the interaction between age and WMH burden (*b* = –0.010, 95% CI [–0.024, .004], *t* = −1.1454, *p* = 0.147). Interestingly, the moderation effect of hypertension on the interaction between different categories of age and WMH burden was statistically significant, revealing intriguing patterns. In the moderation analysis examining the relationship between age and WMH burden, with hypertension as the moderator, using the middle‐aged group as the reference category revealed a significantly negative moderation effect for elderly adults. This suggests that the influence of hypertension on the burden of WMH is less pronounced in elderly adults compared to middle‐aged individuals (*b* = –0.882, 95% CI [−1.591, –0.172], *t* = −2.442, *p* = 0.015). Meanwhile, the interaction with older adults showed positive moderation, though not statistically significant (*b* = .135, 95% CI [–0.140, .410], *t* = .964, *p* = 0.335).

The mediation analysis results provide insights into the relationship between age and WMH burden, with diastolic BP and systolic BP as mediators. The direct positive effect of age on WMH burden was significant, with an effect size of 1.906 (SE = 0.195, *t* = 9.773, 95% CI (1.523 to 2.289), *p* < 0.001), indicating a strong positive association between age and WMH burden, independent of the mediators. The mediation effect of diastolic BP is significant and negative, with an effect size of −0.363 (SE = 0.129, 95% CI (−0.632 to −0.121), *p* < 0.001), suggesting that diastolic BP is associated with a reduction in the impact of age on WMH burden. The mediation effect of systolic BP is significant and positive, with an effect size of 0.468 (SE = 0.106, 95% CI (0.275 to 0.693), *p* < 0.001), indicating that systolic BP is associated with an increase in the impact of age on WMH burden.

The statistical outcome from moderation and mediation analysis is summarized in Table 5.

**TABLE 5:** Moderation and mediation analysis.

Moderation analysis and mediation analysis						
1. Model Summary for moderation (age not categorized)						
R	R‐sq	MSE	F	df1	df2	*p*
0.499	0.249	0.469	55.682	3.000	504.000	0.000
	Coeff	Se	*t*	LLCI	ULCI	*p*
Constant	−1.877	0.335	−0.5.597	−0.25336	−1.218	<0.001
Age	0.044	0.005	8.277	0.033	0.054	<0.001
Hypertension	0.988	0.443	2.229	0.117	1.859	0.026
Int_1^*^	−0.010	0.007	−1.454	−0.024	0.004	0.147

2. Model Summary for moderation with age categorized.						
R	R‐sq	MSE	F	df1	df2	*p*
0.450	0.202	0.500	25.472	5.000	502.000	0.000
	Coeff	Se	*t*	LLCI	ULCI	*p*
Constant	0.537	0.078	6.873	0.383	0.690	<0.001
Older adults	0.452	0.095	4.757	0.265	0.638	<0.001
Elderly adults	1.797	0.299	6.009	1.209	2.384	<0.001
Hypertension	0.282	0.117	2.409	0.052	0.511	0.016
Int_1	0.135	0.140	0.964	−0.140	0.410	0.335
Int_2	−0.882	0.361	−2.442	−1.591	−0.172	0.015

3. Effect of age on WMH severity in blood pressure mediation analysis						
	Effect	Se	*t*	LLCI	ULCI	*p*
Direct effect by age	1.906	0.195	9.773	1.523	2.289	<0.001
Mediation effect by DBP	−0.363	0.129	n/a	−0.632	−0.121	<0.001
Mediation effect by SBP	0.468	0.106	n/a	0.275	0.693	<0.001

*Note*: Given that the **middle‐aged group** is the **reference category**, HTN = hypertension, SDB = systolic blood pressure, DBP = diastolic blood pressure, LLCI and ULCI respectively = lower and upper limit confidence intervals. Int_1^*^ = interaction between hypertension and WMH severity across non‐categorized ages. Int_1 = interaction between HTN and WMH severity among older adults. Int_2 = interaction between HTN and WMH among elderly adults.

## 4 Discussion

This study examined how the severity of WMH varies with age in both stroke patients and individuals without stroke, and explored how modifiable risk factors, particularly hypertension, affect the burden of WMH in different age groups. The study also elucidated the mediation effects of BP on the relationship between aging and WMH burden. The findings illuminate the complex relationship between WMH and cerebrovascular risk factors, providing new insights into how these factors modulate the severity of WMH across different ages, both in individuals with and without a history of stroke.

Our investigation into the prevalence and severity of WMH has uncovered a complex picture dubbed “similar prevalence but different severity” that challenges the conventional understanding of WMH distribution between stroke and normative populations. There is a marked 77.8% incidence of WMH occurrence across our combined cohort, and this prevalence is similarly shown among stroke and stroke‐free normative participants. This finding suggests that the mere presence of WMH is not solely associated with stroke and that asymptomatic stroke‐free individuals are equally at risk [4]. However, the critical distinction between each population emerges when considering the severity of WMH. While the prevalence of WMH may be similar, the severity burden of WMH differs, with higher burdens noted among stroke patients. In their investigation to test if the location of small vessel lesions could explain the acute symptomatic and silent small vessel lesions, Hernández and colleagues [37] made similar assertions that confirm this WMH differential severity. Our findings also support the earlier research by Wei and colleagues [38], who found that compared to healthy controls, stroke patients had much more white matter lesions in almost every area of the brain, pointing to higher burdens of severity.

This study further provides new insights into the distribution of these manifestations across middle‐aged, older, and elderly adults. WMH was less prevalent among middle‐aged adults as compared to older and elderly adults. WMH was predominantly observed among older adults aged 60–79 years, compared to both elderly and middle‐aged adults, a trend consistently evident in both the normative and stroke participant cohorts. However, it was the elderly participants, aged 80–90 years, who exhibited the most severe burdens of WMH. These findings point to an age‐based variation in WMH prevalence and severity among adults. This observation propelled us to delve deeper into the position of hypertension–a factor that, alongside age, emerges as critically significant in our analysis. Consistent with previous studies [37, 39–41], the predictive impact of hypertension across the entire cohort, along with the notably higher incidence of hypertension in the stroke cohort compared to their normative counterparts, provides compelling empirical evidence explaining the increase in WMH burden. The current findings from the moderation analysis underscore hypertension as a pivotal determinant that may exacerbate the vulnerability of cerebral white matter to structural deterioration, predominantly in aging individuals with a history of stroke. More intriguingly, the modulatory influence of hypertension on the relationship between age and WMH severity burden is not uniform but only discernible within specific age groups. The significantly negative interaction observed in elderly adults, when compared to middle‐aged adults as the reference category, suggests that the effect of hypertension on WMH burden is less pronounced in the elderly group. This implies that although hypertension exacerbates WMH severity with advancing age, its impact is greater in middle‐aged adults (**40**–**59 years)** compared to those of elderly age (**80**–**90 years**). These observations align with the research conducted by Guevarra et al. [39], which suggests that the detrimental effect of hypertension on WMH is more significant in people under 70 years.

More intriguingly, findings from the mediation analysis underscore the complex interplay between age, BP, and WMH burden, highlighting distinct roles for systolic and diastolic BP as mediators. The direct positive effect of age on WMH burden aligns with existing literature that identifies age as a primary risk factor for WMH accumulation [20, 39, 42, 43]. The positive mediation effect of systolic BP indicates that elevated systolic BP exacerbates the impact of age on WMH burden. This finding supports the emphasis on controlling systolic hypertension in older adults to mitigate the progression of cerebral pathological changes and cognitive implications [8, 44]. The exacerbating effect of systolic BP on the WMH burden may be linked to increased arterial stiffness, endothelial dysfunction, and reduced cerebral perfusion [5, 6]. Conversely, the negative mediation effect of diastolic BP suggests a protective role, which may inform therapeutic targets aimed at maintaining optimal diastolic BP levels to reduce WMH burden [45, 46]. Understanding the BP‐related mechanisms can inform therapeutic, preventive, and management strategies to preserve brain health and reduce cognitive decline risk.

These findings further imply that although stroke‐free normative participants exhibit a similar prevalence but milder burdens of WMH compared to stroke patients, their susceptibility to hypertension could lead to a progression from mild to severe WMH burdens if BP is not effectively managed from early to mid‐adulthood. This assertion aligns with similar observations regarding the increased vulnerability of elderly stroke patients to a higher WMH burden associated with hypertension [47]. These outcomes further highlight the fact that the modulating impact of hypertension is age‐specific; hence, targeting the age‐stratified hypertension‐induced patho‐mechanisms may yield more accurate prognostic indicators and therapeutic targets.

This study acknowledges several limitations, including the unequal distribution of age groups. While this distribution may introduce bias with continuous variables, the analysis of such variables was based on averages of measured parameters, which helps mitigate the impact of unequal sample sizes by focusing on central tendencies rather than individual variability. WMH was the sole phenotype of cerebral SVD considered in this study because it is the most prevalent and specific marker of aging‐related cerebral SVD, making it a suitable focus for this study [21]. The inclusion of both stroke patients and stroke‐free normative participants was essential to understanding the distribution of WMH across clinical and community‐based settings, offering a comprehensive view of WMH burden in different contexts. This dual‐cohort approach enhances the study's relevance by capturing variations in WMH associated with both pathological and non‐pathological aging processes. Future research should aim to include more diverse populations and additional cerebral SVD phenotypes to further expand upon these findings.

## 5 Conclusion

The surge in cerebral SVDs, as assessed by WMH, is not an incidence of randomness among the aging population. The prevalence of WMH is similar between stroke patients and stroke‐free participants; however, stroke patients exhibit a more severe WMH burden. In a non‐uniform fashion across different age groups, hypertension is a culprit risk factor for exacerbating WMH burden, and middle‐aged adults are the most vulnerable. While the elevation of systolic BP predisposes adults to brain white matter deterioration, the decline in diastolic BP suggests a protective role. Recognizing hypertension as a modifiable risk factor and understanding the aging‐related changes in BP patterns open avenues for developing age‐specific strategies for the mitigation and management of WMH progression.

## Author Contributions

J. A. A. and X. C. were responsible for conceptualizing the research idea and study design. For data collection and management, statistical analysis, literature review, scientific inspection, manuscript drafting, and revision, all authors contributed equally. X. C., J.H., and L.Z. validated the statistical results.

## Ethics Statement

This study protocol was reviewed and approved by the Ethics Committee of The Hong Kong Polytechnic University, approval number [*HSEARS20210720002*].

## Consent

Written Informed consent was obtained from all participants.

## Conflicts of Interest

The authors declare no conflicts of interest.

## Supporting information



Supporting Information

## Data Availability

The data supporting the findings of this study are not publicly available because they contain information that could compromise the privacy of research participants. However, these data can be made available by the corresponding authors, (X.C.), at fiona.chen@polyu.edu.hk, and (J.H.) at dochj@163.com, upon reasonable request.
