# Supplementary material for: Modulatory Effects of Hypertension on Aging‐Related White Matter Hyperintensities: A Comparative Study Among Stroke Patients and Stroke‐Free Community‐Based Cohort
Source: J Clin Hypertens (Greenwich). 2025 Feb 28;27(3):e70002. doi: 10.1111/jch.70002 (PMC11870747; doi:10.1111/jch.70002)
Supplement: Supplementary file 1 — Supporting Information [file JCH-27-e70002-s001.docx]

Supplementary table

Test for collinearity between age and hypertension

| *Coefficients^a^* | | | | | | | | | | |
| --- | --- | --- | --- | --- | --- | --- | --- | --- | --- | --- |
| Model | | Unstandardized Coefficients | | Standardized Coefficients | t | Sig. | 95.0% Confidence Interval for B | | Collinearity Statistics | |
|  |  | B | Std. Error | Beta |  |  | Lower Bound | Upper Bound | Tolerance | VIF |
| 1 | (Constant) | -3.712 | .510 |  | -7.282 | <.001 | -4.713 | -2.710 |  |  |
|  | Age | .080 | .008 | .392 | 10.017 | <.001 | .064 | .095 | .996 | 1.004 |
|  | Hyper-tension | .906 | .142 | .250 | 6.392 | <.001 | .627 | 1.184 | .996 | 1.004 |
| a. Dependent Variable: WMH severity scores | | | | | | | | | | |
